# Tumor Vessel Normalization via PFKFB3 Inhibition Alleviates Hypoxia and Increases Tumor Necrosis in Rectal Cancer upon Radiotherapy

**DOI:** 10.1158/2767-9764.CRC-24-0077

**Published:** 2024-08-09

**Authors:** Marcus Edelmann, Shuang Fan, Tiago De Oliveira, Tina Goldhardt, Dorothée Sartorius, Teona Midelashvili, Karly Conrads, Niels B. Paul, Tim Beißbarth, Johannes R. Fleischer, Moritz L. Blume, Hanibal Bohnenberger, Natasa Josipovic, Argyris Papantonis, Michael Linnebacher, Leif H. Dröge, Michael Ghadimi, Stefan Rieken, Lena-Christin Conradi

**Affiliations:** 1 Department of General, Visceral and Pediatric Surgery, University Medical Center Göttingen, Göttingen, Germany.; 2 Department for Medical Bioinformatics, University Medical Center Göttingen, Göttingen, Germany.; 3 Institute for Pathology, University Medical Center Göttingen, Göttingen, Germany.; 4 Molecular Oncology and Immunotherapy, Department of General, Visceral, Vascular and Transplantation Surgery, University of Rostock, Rostock, Germany.; 5 Department of Radiotherapy and Radiation Oncology, University Medical Center Göttingen, Göttingen, Germany.

## Abstract

Treatment of patients with locally advanced rectal cancer (RC) is based on neoadjuvant chemoradiotherapy followed by surgery. In order to reduce the development of therapy resistance, it is necessary to further improve previous treatment approaches. Recent *in vivo* experimental studies suggested that the reduction of tumor hypoxia by tumor vessel normalization (TVN), through the inhibition of the glycolytic activator PFKFB3, could significantly improve tumor response to therapy. We have evaluated *in vitro* and *in vivo* the effects of the PFKFB3 inhibitor 2E-3-(3-pyridinyl)-1-(4-pyridinyl)-2-propen-1-one (3PO) on cell survival, clonogenicity, migration, invasion, and metabolism using colorectal cancer cells, patient-derived tumor organoid (PDO), and xenograft (PDX). 3PO treatment of colorectal cancer cells increased radiation-induced cell death and reduced cancer cell invasion. Moreover, gene set enrichment analysis shows that 3PO is able to alter the metabolic status of PDOs toward oxidative phosphorylation. Additionally, *in vivo* neoadjuvant treatment with 3PO induced TVN, alleviated tumor hypoxia, and increased tumor necrosis. Our results support PFKFB3 inhibition as a possible future neoadjuvant addition for patients with RC.

**Significance:**

Novel therapies to better treat colorectal cancer are necessary to improve patient outcomes. Therefore, in this study, we evaluated the combination of a metabolic inhibitor (3PO) and standard radiotherapy in different experimental settings. We have observed that the addition of 3PO increased radiation effects, ultimately improving tumor cell response to therapy.

## Introduction

Colorectal cancer (CRC) is the third most common cancer diagnosed worldwide and the second leading cause of cancer death (1). When further stratified, the worldwide mortality of patients with colon and rectal cancer (RC) accounts to approximately 500,000 and 310,000 cases yearly, respectively (1). Treatment of patients with locally advanced RC is based on neoadjuvant chemoradiotherapy (CRT) followed by conventional surgery. Patients’ response to preoperative radiotherapy (RT) is heterogeneous and ranges from resistance and disease progression despite intensive treatment to complete regression of the tumor (clinical complete response). The extent of tumor regression correlates with the long-term clinical outcome (2, 3). There are several studies assessing the benefits of including patients who respond fully to CRT in *watch-and-wait* strategies showing a striking rate of organ preservation warranting further prospective validation (4, 5). However, a relevant number of patients develop tumor regrowth, and this concept should be further evaluated within prospective clinical trials. It is already well known that the content of oxygen within the tumor tissue plays a crucial role during RT (6, 7). Radiation-resistant cells are found particularly in hypoxic regions; therefore, it is not surprising that solid tumors with high oxygenation show a significantly better response to RT than those that are composed of extensive hypoxic areas (8, 9). Critically, advanced tumors, in particular, show large hypoxic areas, and in general, tumor hypoxia favors the development of resistance to RT and is associated with a poor clinical prognosis (10). Oxygen supply is directly influenced by a structurally and functionally abnormal tumor blood vessel system (11). Abnormal neoangiogenesis and increased glucose metabolism are among the hallmarks of cancer and are therefore of particular interest for the development of new treatment approaches in cancer therapy (9, 12, 13). In the center of this complex environment, blocking of the bifunctional enzyme 6-phosphofructo-2-kinase/fructose-2,6-biphosphatase 3 (PFKFB3), by 2E-3-(3-pyridinyl)-1-(4-pyridinyl)-2-propen-1-one (3PO), seems to be a promising therapeutical approach. As an indirect stimulator of glycolysis, PFKFB3 influences the glycolytic activity of cancer and tumor endothelial cells (TEC) and is therefore involved in rapid vasculature growth and subsequent tumor vessel abnormalities (14, 15). By restoring functional tumor blood vessels, the oxygenation of tumors can be increased, thereby reducing hypoxia. The increased perfusion of the tumor could cause an enhanced formation and fixation of DNA-damaging oxygen radicals (ROS) during radiotherapy as well as improve the distribution of drugs in the tumor tissue (16, 17). Hence, the use of 3PO as a PFKFB3 inhibitor could assist (C)RT by reversing the metabolic state of tumor and endothelial cells, thereby promoting tumor vessel normalization (TVN) to improve oxygenation and radiotherapy efficacy. A large number of tumor cells are characterized by increased PFKFB3 expression, as it is the case in CRC (18), making PFKFB3 an attractive target for the therapy of RC.

In this study, we show that RC treatment could be complemented by the addition of 3PO to its treatment regime. Treatment of colorectal cancer cells increased radiation-induced cell death and reduced cancer cell invasion. Additionally, *in vivo* neoadjuvant treatment with 3PO induced TVN and reduced RC hypoxia. Our findings suggest that the use of 3PO in combination with standard CRT might be a potential therapeutical approach for CRC treatment.

## Material and Methods

### Cell culture

Human CRC cell lines HCT-116 (RRID:CVCL_1729), HT-29 (CVCL_0320), SW-1463 (RRID:CVCL_1718), and SW-837 (RRID:CVCL_1729) were obtained from the American Type Culture Collection (Manassas, USA) and authenticated by short tandem repeat sequencing (DSMZ, Leibnitz-Institute, Braunschweig, Germany). CRC cell lines HCT-116 and HT-29 were cultured in McCoy’s medium supplemented with 10% fetal bovine serum (FBS; Biochrom, Berlin, Germany) and 1% Pen/Strep (Gibco, Thermo Fischer Scientific, Waltham, USA) and kept at 37°C with humidified 5% CO_2_ incubators. SW-1463 and SW-837 cells were cultured with Leibovitz’s L-15 medium supplemented with 10% FBS (Biochrom) and 1% Pen/Strep (Gibco) and stored at 37°C with humidified atmospheric air incubators. Human retinal epithelial cells (RPE-1, RRID:CVCL_4388) were kindly provided by Dr. Holger Bastians (Institute for Molecular Oncology, University Medical Center Göttingen, Germany), authenticated by short tandem repeat sequencing (DMSZ, Leibnitz-Institute, Braunschweig, Germany), cultured in DMEM:F12 medium (Gibco), supplemented with 10% FBS (Biochrom) and 1% Pen/Strep (Gibco), and kept at 37°C with humidified 5% CO_2_ incubators. Human mammary epithelial cells (hTERT-HME-1, RRID:CVCL_3383) were provided by Dr. Claudia Binder (Clinic for Hematology and Medical Oncology, University Medical Center Göttingen, Germany), cultured in mammary epithelial cell growth media kit (PromoCell, Heidelberg, Germany), supplemented with 10% FBS (Biochrom) and 1% Pen/Strep (Gibco), and kept at 37°C with humidified 5% CO_2_ incubators. Primary, single-donor human umbilical vein endothelial cells (HUVEC) were isolated and provided by Dr. Joanna Kalucka (Aarhus Institute of Advanced Studies, Aarhus University, Aarhus, Denmark) and cultured at 37°C with 5% CO_2_ humidified incubators with a 1:1 v/v mixture of Endopan 300 SL medium (PAN Biotech, Aidenbach, Germany) and Medium 199 (Gibco) supplemented with 2 mL of endothelial cell growth supplement (ECGS/heparin; PromoCell). Final FBS concentration in the media was 10%. Cells between passages 5 and 10 (HCT-116, HT-29, SW-1463, SW-837, HME-1, and RPE-1) and between passages 2 and 3 (HUVECs) were used for experiments. All cell lines used were negative for mycoplasma contamination, and experiments were performed under normoxic conditions.

### xCELLigence assay

xCELLigence assay was performed as previously described (18). Shortly, by measuring the impedance of bottom coated plates with microelectrodes, the xCELLigence software provides a method to analyze cell proliferation, adhesion, morphology, and death in real time. Changes in cell size, cell number, and cell adhesion result in changes of impedance, which are reflected as cell index (CI). Every condition was tested in triplicates, and the assay was performed three times. Conditions were compared by fitting a saturation curve in logarithm and predicting the 80-hour time point which was then compared using a *t* test.

### CellTiter-Blue assay

The assay was done as previously described (18), following the manufacturer’s protocol (Promega, Madison, USA). Initially, 1 × 10^4^ (HCT-116, HT-29, SW-1463, SW-837, RPE-1) or 1.5 × 10^4^ (HUVEC) cells/well were seeded into 96-well black polystyrene microplates (Corning, NY, USA), resulting in approximately 70% cellular confluence. Reduced resazurin (Resorufin) was measured using the VICTOR Multilabel Plate Reader (PerkinElmer, Inc., Waltham, MA, USA) at 560ex/590em nm wavelength. Every condition was tested in triplicates, and the assay was performed three times. Cell viability is shown as a percentage of the control.

### Lactate dehydrogenase assay

Lactate dehydrogenase (LDH) Cytotoxicity Detection Kit-PLUS (Roche, Diagnostics, Mannheim, Germany, RRID:SCR_025096) was used according to the manufacturer’s protocol. Briefly, 7.5 × 10^3^ (HCT-116, HT-29, SW-1463, SW-837), 1 × 10^4^ (RPE), or 1.5 × 10^4^ (HUVECS) cells/well were seeded into 96-well-plates (Corning, Corning, NY, USA), resulting in approximately 60% to 70% cellular confluence. The absorbance of formazan salt was read using the VICTOR Multilabel Plate Reader spectrophotometer (PerkinElmer, Inc., Waltham, MA, USA) at a 490-nm filter. Every condition was tested in triplicates, and the assay was performed three times.

### Invasion assay

The invasion assay was performed using a Boyden chamber, coated with Cultrex Basement Membrane Extract, PathClear (R&D Systems, MN, USA), as described previously (19). 1 × 10^5^ cells were seeded into the upper well of the Boyden chamber in a 1,000 µL medium. 3PO was added into both sides of the chamber. For the invasion assay in combination with irradiation, CRC cells were irradiated in 75-mL cell culture flask using RS 225 X-ray irradiation system (200 kV, 15 mA, 0.5 mm Cu filter, Gulmay Medical System, Xstrahl Ltd., Camberley, Surrey, UK) with a single dose of 6 Gy. Next, irradiated cells were seeded into the Boyden chamber. After 96 hours of incubation at 37°C and 5% CO_2_, the medium from the upper chamber was removed, and the content of the lower part, containing the invaded cells (both floating and adherent cells), was collected. Cells were centrifuged at 500 *g* for 5 minutes, stained with 0.4% Trypan Blue (Carl Roth, Karlsruhe, Germany) and counted manually using a Neubauer-improved chamber (Paul Marienfeld GmbH, Lauda-Königshofen, Germany). Every condition was tested in triplicates, and the assay was performed three times.

### Migration assay

Migration assay was performed as previously described (18), with cells previously incubated in 0.5 µmol/L mitomycin C for 4 hours. Silicone inserts (ibidi Integrated Biodiagnostics, Germany) were removed when cell confluency was reached (100%). Migration assay in combination with irradiation was performed using RS 225 X-ray irradiation system (200 kV, 15 mA, 0.5-mm copper filter, Gulmay Medical System, Xstrahl Ltd., Camberley, Surrey, UK), 4 hours after cells were exposed to 3PO with a single dose of 6 Gy. Analyses were performed using ImageJ 1.43u software (National Institutes of Health, Bethesda, Maryland, USA. 1997–2016) and GraphPad Prism 9 (GraphPad Software, Inc., La Jolla, CA, RRID:SCR_002798). Migration was calculated with the following formula: migration (%) = A0/Ax. Changes of the area of the gap (Ax) were set in relation to the area of the gap at a time of 0 hour (A0). The resulting percentage describes the migration ability of the cells.

### Spheroid formation assay

For spheroid formation, 5 × 10^5^ HUVEC cells were mixed with 10-mL Endothelial Cell Growth Medium 2 (ECGM2, PromoCell, Heidelberg, Germany, RRID:SCR_023579) supplemented with 2% v/w sterile methylcellulose (Sigma-Aldrich, St. Louis, USA). Tthe cell suspension (25 µL) was turned upside down (hanging drops) for overnight incubation (approx. 16 hours). The next day, spheroids were gently washed and collected into a 50-mL conical tube with approximately 10 mL of PBS (Gibco) supplemented with 10% FBS (Biochrom). After 5 minutes of centrifugation at 300 *g* at RT with no brakes, ECGM2 medium (PromoCell) supplemented with 5 mg/mL fibrinogen (Sigma-Aldrich) was gently overlayed on the pellet and mixed. For solidification, 0.6 U (approx. 6 µL/mL) thrombin (Sigma-Aldrich) was added and again gently mixed. Approximately 500 µL of the mixture-containing spheroids was platted into 24-well plates (SARSTEDT, Hildesheim, Germany). Spheroids were cultured for 24 hours, with medium containing DMSO (control) or with 3PO. After 24 hours, cultures’ images were captured, and *in vitro* angiogenesis was quantified digitally by measuring the length and number of the sprouts (calculated as cumulative sprout length). For the treatment of well-stablished spheroid sprouts, we have allowed sprouts to form for 24 hours and then add 3PO in different concentrations into the medium for another 24 hours. Images were analyzed using the ImageJ 1.53a software (National Institutes of Health, Bethesda, Maryland, USA. 1997–2016). At least five spheroids per each independent experiment were analyzed.

### RT-PCR

RNA extraction and real-time PCR analysis were performed as recently described (18).

### Patient-derived organoids and Western blot analysis

Fresh tissue samples used in this study were provided by the University Medical Center Göttingen (UMG), Germany, after surgical tumor resection (ethical approval UMG Antragsnr. 25/3/17, in line with the ethical principles of the Declaration of Helsinki).

Intestinal patient-derived organoids were established and cultured as previously described (18). For RNA sequencing analysis, three right-sided colon tumors were used. PT6 (colon ascendens), PT12 (coecum), and PT16 (ascendens, right colic flexure) were all at their second passage. Organoids’ medium was supplemented with 30 µmol/L 3PO, and its effects were evaluated after 24 hours by light microscopy. Size quantification was performed using ImageJ 1.43u software (National Institutes of Health, Bethesda, Maryland, USA. 1997–2016, RRID:SCR_003070). Western blot analysis was performed as previously described (18), with three different treatment-naïve rectal tumor organoids (PT70, PT73, and PT79) and HCT-116, HT-29, SW-1463, and SW-837 colorectal cancer cells. The following primary antibodies were used: NDUFB6 (1:1,000, RRID:AB_2235901), ACTIN (1:5,000, RRID:AB_2782959), and ACAD9 (1:500, RRID:AB_2241997), all from Proteintech Germany GmbH, Planegg-Martinsried, Germany; LDHA (1:1,000, RRID:AB_2137173) from Cell Signaling Technology, Massachussets, USA; HSC70 (1:1,000, RRID:AB_627761) from Santa Cruz Biotechnology, Heidelberg, Germany; and PFKPB3 clone MA5-32766 (1:1,000, RRID:AB_2810043) from Invitrogen. After a 60 minutes of incubation with their respective HRP-conjugated secondary antibodies (Invitrogen), membranes were developed using the SuperSignal West Pico/Fento chemiluminescence substrate (Thermo Fischer Scientific). Immunoblots were quantified using ImageJ 1.43u software (National Institutes of Health, Bethesda, MD, USA).

### RNA sequencing

3PO-treated organoids were harvested in TRIzol LS (Life Technologies, RRID:SCR_008817). Total RNA was isolated and DNase I treated using the Direct-zol RNA Miniprep kit (Zymo Research, RRID:SCR_008968) according to the manufacture’s protocol. The sequencing was performed as previously published (20), following a modified nonstrand-specific, massively parallel cDNA sequencing (RNA-seq) protocol from Illumina (Illumina, San Diego, USA, RRID:SCR_010233) starting with 200 ng RNA. The TruSeq RNA Library Preparation Kit v2, Set A (48 samples, 12 indexes; Illumina) was used. The quality and integrity of RNA were assessed via the Fragment Analyzer (Agilent, Sta. Clara, USA, RRID:SCR_019411; RIN >8), and the dsDNA 905 Reagent Kit (Agilent) exhibiting an average size of 260 bp was used to determine the size of final cDNA libraries. Libraries have been pooled and sequenced on Illumina HiSeq 4000 (Illumina, RRID:SCR_016386), which generates 50-bp single-end reads (30–40 Mio reads/sample). mRNA sequencing data analysis was performed according to Sofiadis and colleagues (21). Alignment of the raw reads to the humane reference genome (hg19) was performed using the STAR aligner software (version 2.7.3a, RRID:SCR_004463; ref. 22), in default settings. For quantification, the *featureCounts program* (version 2.0.0, RRID:SCR_012919; ref. 23) was used. To correct for unwanted variations, the RUV function of the RUVseq software (version 3.13, RRID:SCR_006263; ref. 24) was applied before differential gene expression was analyzed by DESeq2 package (version 3.13, RRID:SCR_000154; ref. 25). The RNA sequencing data has been deposited at Gene Expression Omnibus under the accession number GSE270210.

### Gene set enrichment analysis

Gene set enrichment analysis software (GSEA, University of California San Diego and Broad Institute, USA, RRID:SCR_003199; version 4.1.0; ref. 26) was used to analyze the gene expression data at the level of gene sets to show significant differences between the phenotypes tumor control and tumor treated with 30 µmol/L 3PO. Genes were ranked in a list according to expression differences between the respective classes. The ranking metric used was signal-to-noise ratio. GSEA calculates an enrichment score that displays the level of overrepresentation of a defined set of genes at the top or bottom of the ranked list. The ES is normalized with respect to differences in gene set size creating the normalized enrichment score. A false discovery rate cutoff of 5% was used since the permutation type was set to “gene set” because less than seven samples were analyzed per condition. The number of permutations was set to 1,000. The collection “Biological Process” (c5.go.bp.v7.4.symbols.gmt.txt) contributed by Gene Ontology (Gene Ontology Consortium; ref. 27) was downloaded from the GSEA website as part of the Molecular Signature Database (MSigDB; ref. 28).

### Colony formation assay

CRC cells were seeded into six-well plates (Corning, NY, USA) at a density depending on the irradiation dose (further details on request) and incubated at 37°C. After 24 hours of incubation, the medium was replaced by medium containing 2.5 µmol/L 3PO for the combined treatment. Irradiation was performed 4 hours after cells were exposed to 3PO with a dose of 0, 1, 2, 4, 6, and 8 Gy. The plates were incubated at 37°C for 6 days for HCT-116, 7 days for HT-29, 14 days for SW-1463, and 14 days for SW-837 cells. Next, cells were fixed with 70% ethanol and stained with crystal violet. The colonies formed was determined under a stereomicroscope. Colonies containing >50 cells were counted as survivors. Survival fractions were calculated using the following formula: (number of colonies/number of cells plated) irradiated/(number of colonies/number of cells plated) untreated. Data were fitted to the cell survival curve according to a linear quadratic model using GraphPad software (La Jolla, CA, USA, RRID:SCR_002798). The coefficient of drug interaction (CDI) was taken to analyze the interactions among 3PO and radiation therapy. In accordance with the CDI values, the interactions were considered as synergistic, additive, or antagonistic. The CDI was computed based on the following formula: CDI = AB/(A × B), whereby AB is the ratio of the combined treatment to the control group and A/B is the ratio of the single treatment modality to control group. A CDI value <1, = 1, or >1 represents that a combination of 3PO and radiation therapy is synergistic, additive, or antagonistic, respectively (29). The experiments were performed in triplicates and independently repeated three times.

### Establishment of patient-derived xenografts and radiation treatment

NOD.CB17-Prkdcscid/Rj mice (Janvier Labs, Le Genest-Saint-Isle, France, RRID:IMSR_RJ:NOD-SCID) animals were housed at the UBFT animal facility from the University Medical Center Göttingen, under pathogen-free conditions. Mice were kept in individually ventilated cages (IVC), in standardized conditions at 23°C ± 1°C RT, and in controlled 12-hour light/dark cycle. Males and females (9 weeks old) were used in the experiments. Food and water were provided *ad libitum*. For implantation, approximately 2 mm^3^ rectal tumor fragments (PDX HROC239, primary moderately differentiated rectal adenocarcinoma: G2, pT4b pN2b (7/20) L0 V0 Pn1 cM0) were subcutaneously engrafted into the flank of mice. Tumor growth was measured three times per week with a caliper, and tumor volumes were estimated using the following formula: [length (larger axis, in mm) × width (smaller axis, in mm)^2^]/2. When primary outgrowths reached a total volume of 1,000 mm^3^, tumors were explanted, and fragments were retransplanted into new hosts as secondary xenografts (F1). Patient-derived xenografts after two retransplantations (F2) were used for therapy approaches. When implanted tumors reached an average volume of ∼100 to 200 mm^3^, mice were randomized and treated. The radiotherapy cohort mice received either vehicle (DMSO; *n* = 5), an intraperitoneal injection of 3PO (25 mg/kg, every three 3 days for three consecutive days; *n* = 4), irradiation (*n* = 4), or combined therapy of 3PO and irradiation (*n* = 5).

For irradiation, mice were first anesthetized with approximately 5% sevoflurane (Baxter; 1l O_2_/minutes) and then placed on a special designed cage where the delivery of gas anesthesia could be constant at 2.5% to 3% (1l O_2_/minute). Next, animals were covered with a cropped leaded protection, and only the tumors on the flank were exposed and irradiated with an X-ray tube (Gulmay Medical System, Xstrahl Ltd., Camberley, Surrey, UK). The 1.8  Gy (70 kV and 25 mA, 0.5-mm aluminum filter, table distance of 470 mm) daily dose of radiation was delivered 14 consecutive times, reaching a total dose of 25.2 Gy [biological equivalent dose (BED) = 29.78 Gy]. The used radiation dose and its fractionation represent approximately half of the dose applied in patients (30). On Saturdays and on Sundays, no irradiation was administrated to animals. At end of the experiment, tumor weight was registered, and samples were collected for histological examination. All procedures were approved by the Niedersächsisches Landesamt für Verbraucherschutz und Lebensmittelsicherheit (LAVES), Germany (17.2407 and 21.3816).

### Histology and immunostainings

The methods for histology and immunostainings have been described previously (14). Immunostainings were done with the following antibodies: CD-31 (BD Biosciences, 1:500, RRID:AB_394816), BrdU, (Bio-Rad, 1:500, RRID:AB_2922993), PFKFB3 clone MA5-32766 (Invitrogen, 1:200), αSMA (Cell Signaling Technology, 1:500, RRID:AB_2734735), CLEAVED CASPASE-3 (Cell Signaling Technology, 1:500, RRID:AB_2070042), and γ-H2AX (Cell Signaling Technology, 1:500, RRID:AB_2118009). ProLong Mountant Media with DAPI (Invitrogen) was used for IF samples. Tumor hypoxia was measured as previously described using Hypoxyprobe Kit (Chemicon/Millipore, Merck, Germany; ref. 14). The hypoxic area of the tumor was determined as a percentage of the total viable tumor area. Cell proliferation was detected by BrdU Incorporation (Roche); 60 mg/kg pimonidazole hydrochloride (Hypoxyprobe) and 75 mg/kg BrdU (Sigma) were injected *i.p.* into tumor-bearing mice 90 minutes before tumor harvesting. Pimonidazole positivity in pictures of the histological slides was determined using the “color threshold” tool of the ImageJ 1.43u software (National Institutes of Health, Bethesda, Maryland, USA. 1997–2016). BrdU positivity was calculated dividing the number of BrdU-positive tumor cells by the total number of tumor cells in the sections. Tumor necrosis has been scored on hematoxylin and eosin (H&E)-stained paraffin sections. Therefore, the viable tumor area was quantified in representative H&E images using the ImageJ 1.43u software. Viable tumor regions were marked with the free hand tool, and the resulting area was divided by the overall area of the picture to create an index.

### Terminal deoxynucleotidyl transferase–mediated dUTP nick end labeling assay

Terminal deoxynucleotidyl transferase–mediated dUTP nick end labeling (TUNEL) assay was performed with paraffin sections, using the DeadEnd Fluorometric TUNEL System kit (Promega, Madison, WI, USA), as recommended by the manufacturer’s manual.

### Flow cytometry

For flow cytometry, 3 × 10^5^ cells were seeded in six-well plates and allowed to attach overnight. On the next day, the complete medium was exchanged for 3PO-containing medium in the desired concentrations. After 24 hours of treatment, cells were harvested using trypsin (Gibco; approx. 5–10 minutes at 37°C), washed with 1× PBS, and resuspended in complete medium containing 50 µg RNAse (Sigma). After 10 minutes of incubation on ice, propidium iodate (PI) was added to a final concentration of 50 µg/mL. Cells were kept on ice and immediately analyzed with LSR Fortessa X-20 Cell Analyzer (Becton Dickinson, USA) using a yellow-green laser with 610/20 nm (ex/em). Interpretation of the data was done using Flow Jo 10.8.1 (Becton Dickinson, RRID:SCR_008520). Singlets were gated via size exclusion, and thresholds were designed with the use of single-stained untreated samples. Graphics were produced using GraphPad Prism 9 (GraphPad Software Inc.).

### Seahorse assay

The patient-derived tumor organoid (PDO) was treated with TrypLE (Invitrogen) for 10 minutes at RT, pipetted up and down 10× with a 5-mL serological pipette adapted to a 200 µL pipette tip, and then passed through a 40 µm mash (Greiner Bio One) to obtain a single-cell suspension. Next, cells were manually counted using a Neubauer-improved chamber (Paul Marienfeld GmbH), and approximately 2,000 cells were seeded in 5 µL of Matrigel (Corning) into 96-well spheroid microplates (Agilent Technologies, Santa Clara, CA, USA). Organoids were allowed to grow for 6 days in organoids’ medium before treatment with 10 µmol/L 3PO for 24 hours. Before plate reading, a complete medium was exchanged for 180 µL XF serum-free medium (Agilent) supplemented with 1% sodium pyruvate and glutamine (both from Gibco). Seahorse XFe96 instrument (Agilent, RRID:SCR_019545) was used. All injected reagents (glucose, oligomycin, and 2DG) were from Sigma, and their final concentrations in the medium were 250 mmol/L, 30 µmol/L and 250 mmol/L, respectively. Graphics were produced using GraphPad Prism 9 (GraphPad Software Inc.).

### Statistical analysis

Data are presented as mean ± standard deviation (SD). Statistical analysis was performed with GraphPad Prism 9 (GraphPad Software Inc., RRID:SCR_002798) using two-tailed Student *t* test, unless otherwise indicated in the figure legends. *P* < 0.05 was considered statistically significant. ^∗^, *P* < 0.05; ^∗∗^, *P* < 0.01; ^∗∗∗^, *P* < 0.001; ^∗∗∗∗^, *P* < 0.0001. Samples sizes and number of replicates done are found in the figure legends.

### Data availability

The datasets generated or analyzed as well as all cell lines used during the current study are available from the corresponding author on request.

### Institutional review board statement

The study was conducted according to the guidelines of the Declaration of Helsinki and approved by the Ethical Committee of the University Medical Center, Göttingen (UMG # 25/3/17). A written informed consent has been obtained from all subjects.

## Results

### 3PO affects CRC cell proliferation and viability in a concentration-dependent manner

Previously, the cytotoxic effect of 3PO has already been shown for multiple cancer cell lines (31). However, its impact on colorectal cancer remains unclear. Therefore, to characterize the effect of PFKFB3 blockade on colorectal cancer cells, we decided to work with well-described human cancer cell lines representative of the colon (HCT-116 and HT-29) and rectum (SW-837 and SW-1463; refs. 32, 33).

**Figure 1:**
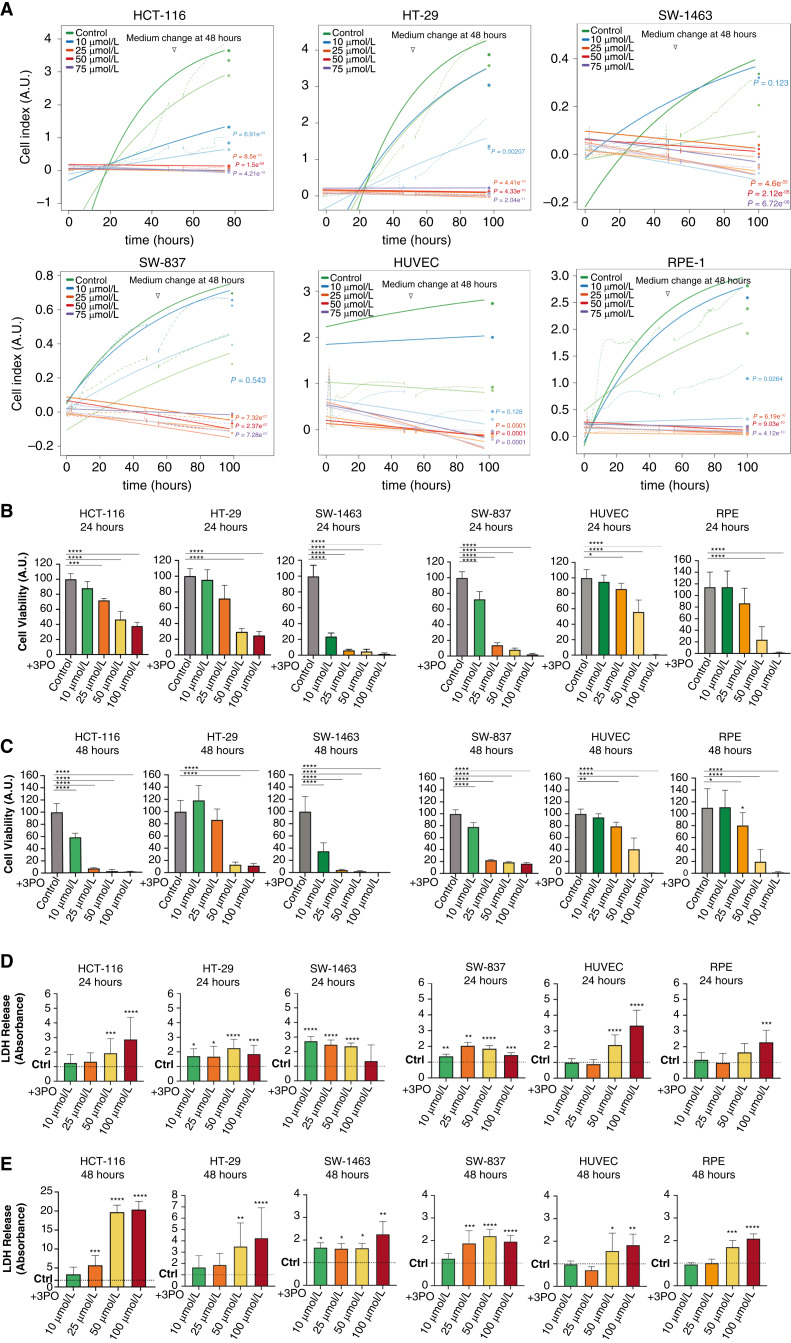
3PO affects normal epithelial and colorectal cancer cell proliferation and viability in a concentration-dependent manner. **A,** xCELLigence assay performed with HCT-116, HT-29, SW-1463, SW-837, HUVECs, and RPE-1 cells. Real-time cell analysis following treatment with different concentrations of 3PO over a period of 96 hours. Dotted lines indicate the fitted saturation curve in logarithm, *n* = 3 independent experiments. **B** and **C,** CellTiter-Blue assay performed with HCT-116, HT-29, SW-1463, SW-837, HUVECs, and RPE cells treated with different concentrations of 3PO for 24 and 48 hours. **D** and **E,** LDH assay performed with HCT-116, HT-29, SW-1463, SW-837, HUVECs, and RPE cells treated with different concentrations of 3PO for 24 and 48 hours. **B–E,** Data are displayed as means SD, ^∗^, *P* < 0.05; ^∗∗^, *P* < 0.01; ^∗∗∗^, *P* < 0.001; ^∗∗∗∗^, *P* < 0.0001. One-way ANOVA was used to calculate statistical significance, *n* = 3 independent experiments.

To evaluate the effects of different 3PO concentrations on HCT-116, HT-29, SW-1463, and SW-837 cancer cells in real time, an xCELLigence assay was performed. Over a period of 96 hours, convergent signaling events, including changes in cell morphology, proliferation, and cell adhesion, were recorded by measuring cellular impedance (CI). We observed that upon treatment with low concentrations of 3PO (10 µmol/L), all cancer cells still maintained their morphological characteristics and proliferative capabilities while showing a minor decrease in CI. However, higher concentrations of 3PO (25, 50, and 75 µmol/L) suppressed proliferation in cancer cells and reduced cell adhesion, resulting in a significant reduction of CI (Fig. 1A). To further evaluate the effects of 3PO on normal, nontransformed human cells, we also subjected endothelial cells (HUVEC), retinal epithelial cells (RPE-1), and mammary epithelial cells (HME-1) to 3PO in an xCELLigence assay. The normal nontransformed cells also maintained their morphological characteristics and proliferative capabilities with low concentrations of 3PO (10 µmol/L) while showing a significant reduction of their cell indexes at higher concentrations of 3PO (25, 50, 75 µmol/L; Fig. 1A; Supplementary Fig. S1A). Additionally, to directly assess cell viability and cell death upon 3PO treatment, we performed CellTiter-Blue assays and LDH-release assays with CRC cell lines as well as with normal, nontransformed cells. A clear time- and dose-dependent reduction of cell viability was observed after 24 hours of treatment with 3PO, and it was intensified after 48 hours (Figs. 1B and C). Corroborating the previous findings, LDH release increased after 24 and 48 hours of treatment with 3PO, indicating cell death is enhanced upon inhibition of glycolysis (Figs. 1D and E). CRC cells showed to be more susceptible to 3PO at lower concentrations (10 and 25 µmol/L) than normal HUVECs and RPE cells. Among CRC cell lines, both RC cell lines SW-1463 and SW-837 exhibited significantly lower cell viability and increased LDH-release compared with the HCT-116 and HT-29 cells. It is noteworthy that immunoblot analysis of the colorectal cancer cell lines’ lysates did not show a direct correlation between PFKFB3 expression and its inhibition’s response by 3PO (Supplementary Fig. S1B).

### 3PO reduces colorectal cancer cell invasion and endothelial sprouting capabilities

**Figure 2:**
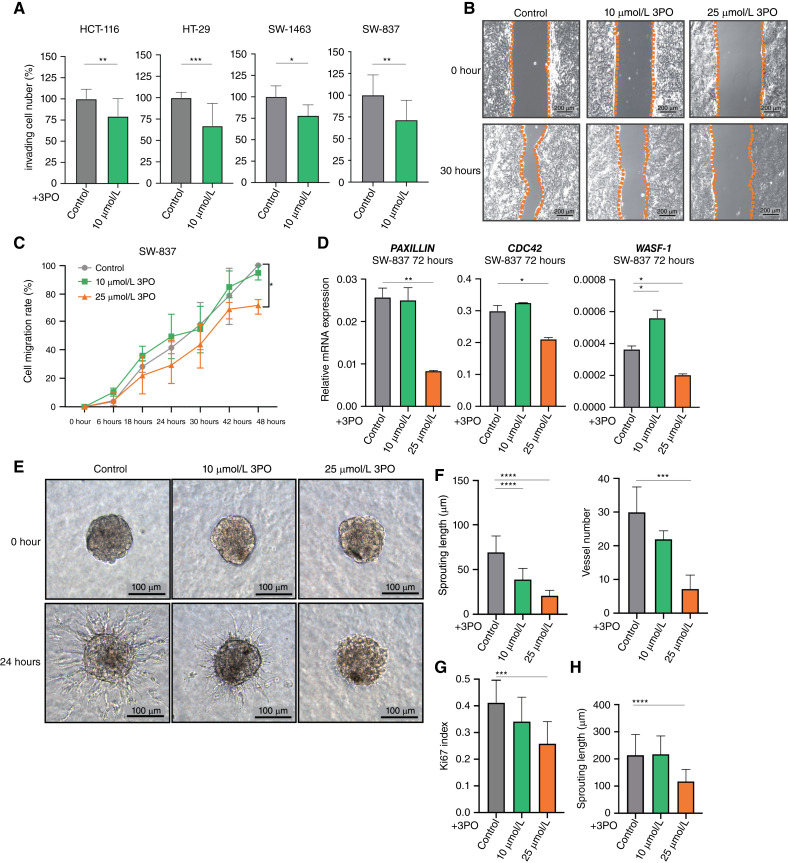
3PO reduces colorectal cancer cell invasion and endothelial cell sprouting capabilities. **A,** Invasion assay performed with HCT-116, HT-29, SW-1463, and SW-837 cancer cells. Cells were plated in a Boyden multi-well chamber and treated with 10 µmol/L 3PO for a period of 96 hours. Data are displayed as means SD, *n* = 3. ^∗∗^, *P* < 0.01; ^∗∗∗∗^, *P* < 0.0001; Student *t* test. **B,** Representative pictures of migration assay performed with SW-837 treated with 10 and 25 µmol/L 3PO; 0 and 30 hours after removing culture inserts. **C,** Cell migration assay quantification of SW-837 treated with different concentrations of 3PO for 48 hours. Data are displayed as means ± SD, *n* = 3. **D,** Relative mRNA expression levels of *PAXILLIN*, *CDC42*, and *WASF-1* in SW-837 cells treated with different concentrations of 3PO after 72 hours. Data are displayed as means SD, ^∗^, *P* < 0.05; ^∗∗^, *P* < 0.01; one-way ANOVA. **E,** Representative pictures of cell sprouting assay performed with HUVECs treated with 10 and 20 µmol/L 3PO after 0 and 24 hours. **F** and **G,** Sprouting assay quantification of HUVECs upon PFKFB3 inhibition. Sprouting length, vessel number, and EC proliferation analyzed upon treatment with 10 and 20 µmol/L 3PO for a period of 24 hours. **H,** Sprouting length quantification upon 24 hours of 3PO treatment on already established spheroids. Data are displayed as means SD, *n* = 3. ^∗∗∗^, *P* < 0,001; ^∗∗∗∗^, *P* < 00,001; one-way ANOVA.

As previously shown, blockade of glycolysis in cancer cells can be associated with cytoskeleton rearrangements, which might impair tumor cell migration as well as tumor cell invasion (18, 34). Accordingly, using a Boyden chamber assay, we evaluated the effects of 3PO on cancer cell invasiveness in HCT-116, HT-29, SW-1463, and SW-837 cells. Here, we found a significant decrease in CRC cell invasiveness using a low concentration of 3PO (10 µmol/L; Fig. 2A). Next, we evaluated 3PO effects on migration by performing a wound healing assay. Time-lapse micrographs and image software analyses were used to monitor the migration of HCT-116, HT-29, and SW-837 cancer cells (Fig. 2B; Supplementary Fig. S1C–S1F). Due to its piled-up growth pattern, SW-1463 cancer cells could not be used for this experimental setup. Though CRC cell lines SW-837 and HT-29 were significantly affected by 25 µmol/L 3PO (Fig. 2C; Supplementary Fig. S1C–S1D), HCT-116 cells showed no changes in migration capabilities (Supplementary Fig. S1E and S1F). To further evaluate the molecular changes in cytoskeleton-related genes, we analyzed the mRNA expression of cell invasion- and migration-related genes. Corroborating our findings, SW-837 and HT-29 cells treated with 3PO had reduced expression of *PAXILLIN*, cell division control protein 42 (*CDC42*), and Wiskott–Aldrich syndrome protein 1 (*WASF-1*), whereas HCT-116 cells did not show relevant changes of the expression of these genes (Fig. 2D; Supplementary Fig. S1G and S1H).

Notoriously, glycolysis controlled by PFKFB3 regulates migration of endothelial cells. In particular, TECs are characterized by an activated glycolytic phenotype and increased expression of PFKFB3 (14, 35). Subsequently, in addition to the motility and invasion effects of 3PO on cancer cells, we also assessed its effects on sprouting and vessel-like formation using endothelial spheroids. Spheroids treated with 3PO showed a significantly shortened sprout length and reduced vessel-like numbers (Figs. 2E and F), in association with reduced proliferation by Ki67 IHC staining (Fig. 2G; Supplementary Fig. S1I). Lastly, to reassess the findings by Kim and colleagues (36) that 3PO only prevents sprout initiation but does not cause the regression of established vascular networks, in our experimental setup, we have performed 3PO treatment on established endothelial sprouts for 24 hours and quantified a significant reduction in sprouting length with 20 µmol/L 3PO (Fig. 2H), corroborating our previous findings *in vivo* (15) and again highlighting model-dependent results.

### Patient-derived cancer organoids treated with 3PO show a gene set enrichment of genes related to oxidative phosphorylation

**Figure 3:**
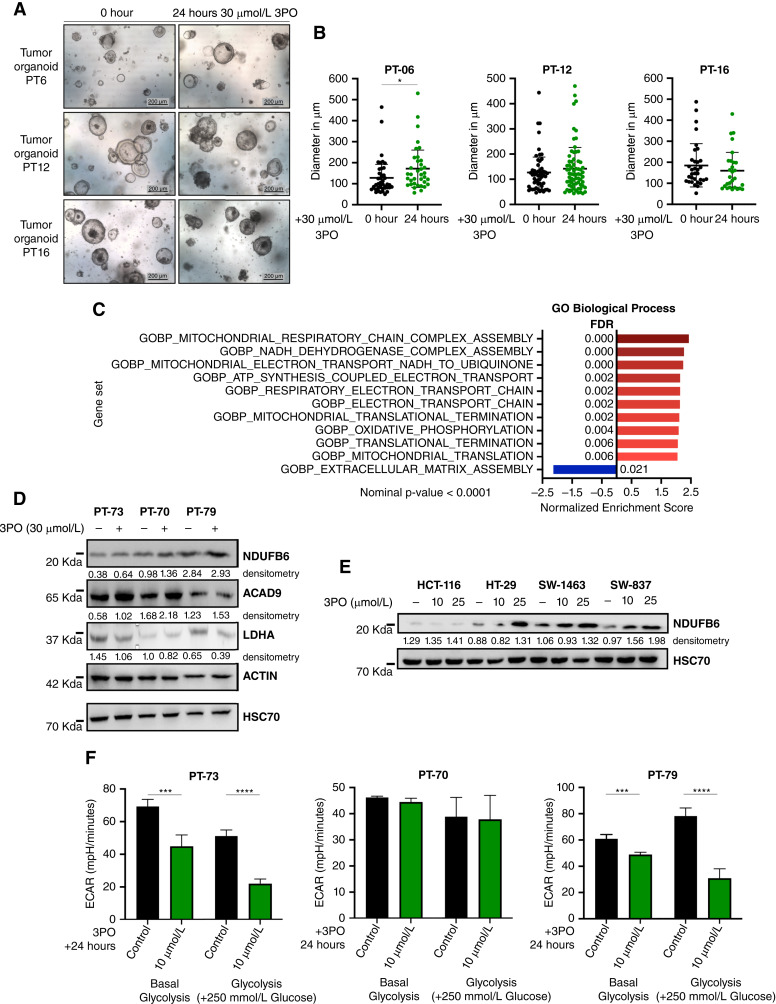
Gene set enrichment analysis of 3PO-treated colon organoids. **A,** Representative pictures of patient-derived tumor organoids treated with 30 µmol/L 3PO for 24 hours. Scale bars, 200 μm. **B,** Patient-derived tumor organoid size quantification. Data are displayed as means ± SD. ^∗^, *P* < 0.05; one-way ANOVA. **C,** GSEA with gene sets derived from the GO Biological Process ontology. For the enrichment plot (score curves), refer to Supplementary Fig. S2. FDR, false discovery rate. **D** and **E,** Protein expression levels of NDUFB6, ACAD9, and LDHA in colorectal cancer organoids (PT-73, PT-70, and PT-79) and colorectal cancer cell lines treated with 3PO for 24 hours. ACTIN and HSC70 were used as loading control. Uncropped Western blot pictures at Supplementary Fig. S5. In all cases, densitometry analysis is performed using loading control as references. **F,** Seahorse analysis for extracellular acidification rate performed with patient-derived tumor organoids treated with 10 µmol/L 3PO for 24 hours. Data is displayed as SD. ^∗∗∗^, *P* < 0.001; ^∗∗∗∗^, *P* < 0.0001.

To evaluate 3PO-induced changes on cellular transcriptomes in a more advanced *in vitro* model, PDOs of three different patients were treated with 30 µmol/L 3PO for 24 hours, and their RNA was extracted and sequenced. As observed in Figs. 3A and B, at the end of this time point, 3PO treatment did not affect tumor organoids’ morphology or growth. Next, the resulting gene expression data were interpreted by GSEA. Supporting the idea that 3PO treatment would influence a metabolic shift away from glycolysis, among the Gene Ontology collection “Biological Process” (27), 9 out of the 10 most enriched gene sets were associated with oxidative phosphorylation and mitochondrial translation (Fig. 3C). In order to evaluate whether these transcriptomic changes were also detectable at the protein level, we further treated three additional different organoids with 30 µmol/L 3PO for 24 hours and collected protein lysates. Immunoblot analyses for the expression of NDUFB6, a gene found enriched in several of the datasets evaluated (Supplementary Fig. S2A–S2C), and ACAD9, a critical influencer on oxidative phosphorylation (37) also found enriched in the GSEA, were also found increased upon 3PO treatment in two of the three PDOs and in three of the four tested colorectal cancer cells (Figs. 3D and E; Supplementary Fig. S3A). Additionally, we were able to see a downregulation of the glycolytic marker LDHA in 3PO-treated PDOs. Last, we treated for 24 hours PDOs with a lower concentration of 10 µmol/L 3PO (to prevent unwanted cytotoxic effects) and performed a seahorse analysis. In line with the previous results, we observed that both the basal glycolysis levels and the glycolysis upon glucose stimulation were reduced in two of the three PDOs. Also, a shift in oxygen consumption ratio was observed in two PDOs treated with 3PO (Fig. 3F; Supplementary Fig. S3B and S3C).

Taken together, these findings suggest that 3PO is able to alter the transcriptomic metabolic status of PDOs toward oxidative phosphorylation and reduced glycolysis.

### 3PO potentializes irradiation effects on colorectal cancer cells

**Figure 4:**
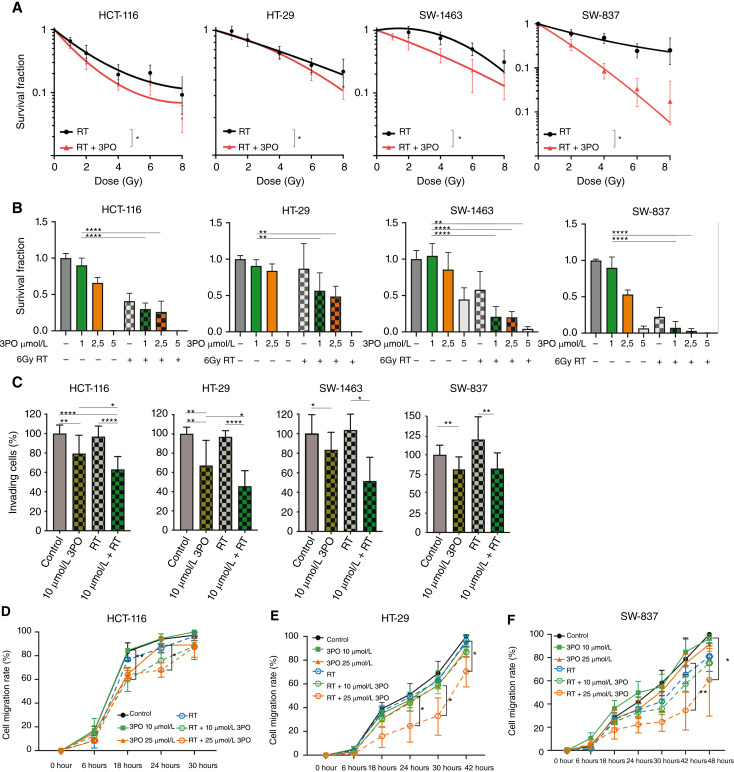
3PO potentializes irradiation effects in colorectal cancer cells. **A,** Colony formation assay performed with HCT-116, HT-29, SW-1463, and SW-837 cancer cells upon 3PO treatment. Cells treated with DMSO or 2.5 µmol/L 3PO for 4 hours and exposed to indicated doses of RT. Six days (HCT-116), 7 days (HT-29), and 14 days (SW-1463 and SW-837) later, colonies were stained and counted. Data are displayed as means ± SD, *n* = 3. ^∗^, *P* < 0.05; one-way ANOVA. **B,** Colony formation assays performed with HCT-116, HT-29, SW-1463, and SW-837 cells treated with different concentrations of 3PO for 4 hours, exposed to either RT (6 Gy) or not irradiated and analyzed after 6 days (HCT-116), 7 days (HT-29), 14 days (SW-1463), and 14 days (SW-837) by counting colonies. **C,** Invasion assay performed with HCT-116, HT-29, SW-1463, and SW-837 cancer cells. Cells were plated in a Boyden multi-well chamber and treated with 10 µmol/L 3PO for 4 hours, subjected to RT (6Gy), and incubated for a period of 96 hours. Data are displayed as means SD, *n* = 3. ^∗∗^, *P* < 0.01; ^∗∗∗∗^, *P* < 0.0001; one-way ANOVA. **D**–**F** Cell migration assay quantification of HCT-116, HT-29, and SW-837 cancer cells upon combinatory treatment. Cells were treated with 3PO for 4 hours and subjected to RT (6 Gy). Data are displayed as means ± SD, *n* = 3. ^∗^, *P* < 0.05; ^∗∗^, *P* < 0.01; ^∗∗∗^, *P* < 0.001; ^∗∗∗∗^, *P* < 0.0001; one-way ANOVA.

Increased glycolysis is crucial for proliferation and survival of tumor cells (38). As a regulator of glycolysis, PFKFB3 plays a substantial role in this metabolic scenario, and a variety of cancer entities have been characterized by enhanced PFKFB3 expression, including CRC. We have previously shown an association of high expression of PFKFB3 with poor survival of patients with CRC (18). Concomitantly, increased glycolysis is assumed to cause resistance to RT and chemotherapy in several cancer types (39). Therefore, we next evaluated the impact of 3PO and radiotherapy combination *in vitro* by a colony formation assay. In all tested colorectal cancer cell lines, a significant decrease in cell survival fraction was observed upon exposure to 3PO (Fig. 4A). The administration of 3PO resulted in a significant reduction of clonogenic survival (which assesses cell death upon treatment with ionizing radiation and the potency of cytotoxic agents) in HCT-116 cells, HT-29 cells, and SW-1463 cells as well as SW-837 cells. To further complement our results, CRC cell lines were subjected to a combination therapy consisting of increasing concentrations of 3PO and a single dose of RT (6 Gy). Corroborating our findings, all CRC cell lines showed a significant decrease in the cell survival fraction when 3PO was added to RT in comparison to 3PO alone (Fig. 4B). In order to provide a more detailed assessment, we calculated the CDI (Supplementary Fig. S4A). According to our results the CDI yielded synergistic interactions across all CRC cell lines (CDI <1). In particular, the lowest CDI values throughout all concentrations were exhibited in RC cell lines SW-1463 and SW-837. Irradiation is well known to alter the biological behavior of tumor cells, and several studies have previously indicated that RT can induce tumor invasion and migration, promoting metastasis (40–42). Thus, we evaluated the combinatory effects of 3PO and RT on cell invasion and migration of CRC cell lines. As shown in Fig. 4C, RT alone had no influence on cancer cell invasion. In line with our previous results (Fig. 2A), invasion of HCT-116 and HT-29 cells was significantly reduced when a low concentration of 10 µmol/L 3PO was combined with RT. However, SW-1463 and SW-837 did not show any significant additional decrease in invasiveness in combination therapy when compared with 3PO alone. Nevertheless, when combined treatment was compared with RT alone, a significant decrease in cell invasion was observed across all CRC cell lines, indicating a favorable impact of 3PO in combination with RT. Next, we performed a wound healing assay combining 3PO and RT. RT alone did not alter the migration behavior of HCT-116, HT-29, and SW-837 cells (Figs. 4D–F; Supplementary Fig. S4B–S4D). Indeed, when RT was combined with 3PO, all CRC cell lines showed a significant suppressed migratory ability (Figs. 4D–F). Furthermore, assessment of HUVEC viability, sprouting, and vessel formation suggested that combining low doses of 3PO to the RT regimen might also affect tumor vascularization (Supplementary Fig. S4E–S4G). In sum, these findings indicate that 3PO is able to enhance radiation effects. The combination treatment produces synergistic effects in CRC cell lines, resulting in decreased cell survival (in all CRC tested cell lines), cell invasion (HCT-116 and HT-29), and cell migration (HCT-116, HT-29, and SW-837), even at a low concentration of 10 µmol/L 3PO analysis.

### 3PO administration in combination with radiotherapy increases tumor necrosis *in vivo* in a rectal patient-derived xenograft mouse model

**Figure 5:**
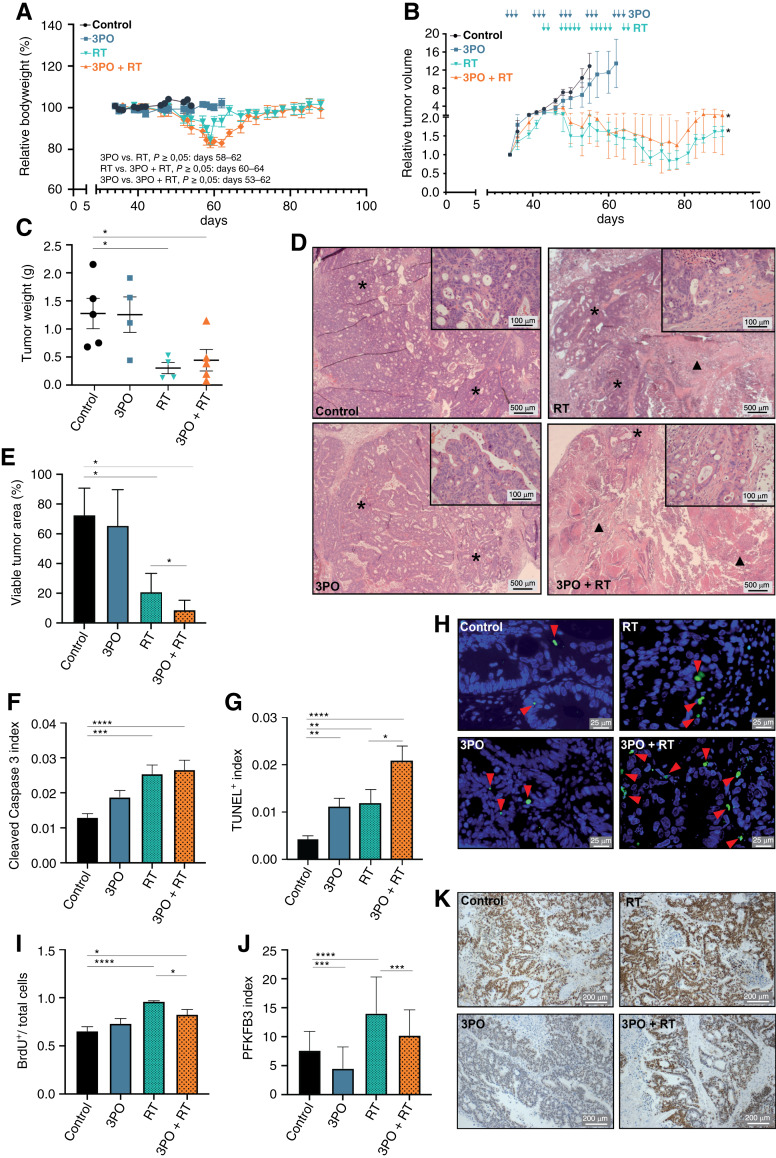
3PO administration in combination with radiotherapy increases tumor necrosis and DNA damage in RC cells *in vivo*. **A,** Relative body weight curves of mice in different therapy groups. **B,** Relative growth curve of rectal PDX tumors upon treatment with vehicle (control; *n* = 5), 3PO (25 mg/kg; 3×/week; *n* = 4), RT (1.8 Gy single dose; 5×/week; *n* = 4), and dual therapy 3PO + RT (25 mg/kg; 3×/week; 1.8 Gy single dose; 5×/week; *n* = 5). Arrows indicate administration of 3PO and RT. Data are displayed as means ± SEM, ^∗^, *P* < 0.05; one-way ANOVA. ^∗^, *P* value refers to RT or RT + 3PO compared with control. **C,** PDX tumor weight. Data are displayed as means ± SD. ^∗^, *P* < 0.05; one-way ANOVA. **D,** Representative H&E staining images of PDX tumors upon treatment with vehicle (control), 3PO, RT, and 3PO + RT. Scale bars, 500 and 100 µm. Arrow heads: representative necrotic areas; asterisks: viable tumor areas. **E,** Viable tumor area quantification (% of total tumor area). Data are displayed as means SD. ^∗^, *P* < 0.05; ^∗∗∗∗^, *P* < 0.0001; Student *t* test. **F,** Quantification of apoptosis by cleaved caspase-3 staining. Data are displayed as means SD. ^∗∗∗^, *P* < 0.001; ^∗∗∗∗^, *P* < 0.0001; one-way ANOVA. **G,** Quantification of DNA fragmentation using TUNEL staining. Data are displayed as means SD. ^∗^, *P* < 0.05; ^∗∗^, *P* < 0.01; ^∗∗∗∗^, *P* < 0.0001; one-way ANOVA. **H,** Representative images from TUNEL staining in PDX tumors upon treatment with vehicle (control), 3PO, RT, and 3PO + RT. Scale bars, 25 µm. **I,** Quantification of proliferation using bromodeoxyuridine (BrdU) staining. Data are displayed as means ± SD. ^∗^, *P* < 0.05; ^∗∗∗∗^, *P* < 0.0001; one-way ANOVA. **J,** Quantification of PFKFB3 expression in tumor tissue. Data are displayed as means SD. ^∗∗∗^, *P* < 0.001; ^∗∗∗∗^, *P* < 0.0001; one-way ANOVA. **K,** Representative images from PFKFB3 staining in PDX tumors upon treatment with vehicle (control), 3PO, RT, and 3PO + RT. Scale bars, 200 µm.

To confirm our results *in vivo*, we established an RC patient-derived xenograft (PDX) model. For this purpose, fresh isolated human rectal tumor samples (RC of the upper third; G2; pT4b pN2b cM0) were transplanted into immunodeficient mice (43) for expansion. Subsequently, upon PDX growth, we followed a treatment protocol that simulated clinical conditions, i.e., fractionated radiation doses (1.8 Gy/dose), in combination with *i.p*. administration of 25 mg/kg 3PO, which has been shown to be well tolerated at this dose and to induce TVN in the B16-F10 mouse model (14). Mouse cohorts that received RT or the combination of RT and 3PO showed significant weight loss during the treatment (Fig. 5A) but no other signs of systemic toxicity. Tumor growth was not suppressed upon treatment with sham (control group) or 3PO alone, and these cohorts had to be sacrificed due to their increased PDX sizes at days 54 and 62, respectively. Both RT alone and 3PO combined with RT cohorts had their tumor growth affected by treatment. However, 3PO and RT in combination did not further reduce tumor growth when compared with RT alone (Fig. 5B).

Tumor regrowth after radiochemotherapy and clinical complete response is an emerging clinical problem (44). Therefore, we sacrificed the two remaining mice cohorts approximately 30 days after the last RT. During this period, none of the cohorts showed sustained regrowth, evidenced by their tumor sizes and tumor weights (Figs. 5B and C). Next, we evaluated histologically all tumor samples, observing that tumors treated with the combination of 3PO and RT had extensive necrotic areas and a difference in tumor viability, when compared with RT-alone tumors (Figs. 5D and E). Although cleaved caspase-3 levels were similar in RT and 3PO in combination with RT cohorts, TUNEL-positive cancer cells were also significantly increased in the combinatory group (Figs. 5F–H). Additionally, we could observe a significant reduction in cell proliferation by BrdU Incorporation, when 3PO was combined with RT (Fig. 5I). Furthermore, PFKFB3 expression levels were found decreased in tumor tissues when 3PO was used alone or in combination with RT (Figs. 5J and K).

Taken together, these results reveal that 3PO administration lowers PFKFB3 expression in tumor tissue, thereby enhancing RT effects, increasing cell death and reducing RC proliferation *in vivo*.

### 3PO administration induces TVN and alleviates hypoxia *in vivo*

**Figure 6:**
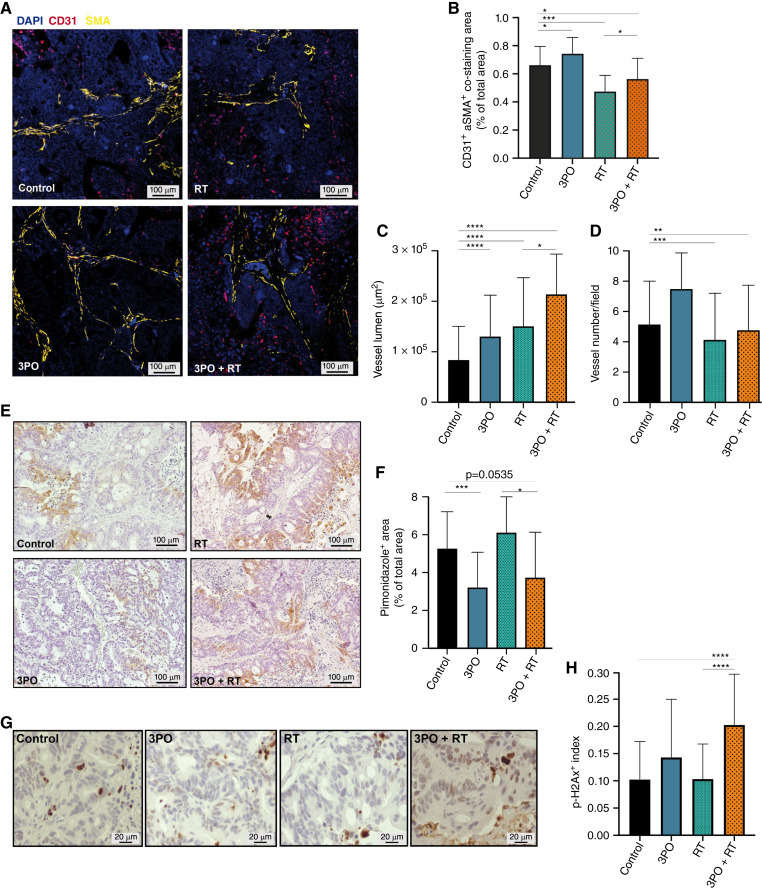
*In vivo* 3PO administration in combination with radiotherapy induces TVN and alleviates hypoxia in RC. **A,** Representative images of CD31^+^αSMA^+^ co-stained sections of PDX tumors upon treatment with vehicle (comtrol), 3PO, RT, and 3PO + RT. Scale bars, 100 μm. **B–D,** Quantification of CD31^+^ αSMA^+^ area (%), vessel lumen size (µm^2^), and tumor vessel number in PDX tumors from control, 3PO, RT, and 3PO + RT-treated mice. Data are displayed as means SD. ^∗^, *P* < 0.05; ^∗∗^, *P* < 0.01; ^∗∗∗^, *P* < 0.001, ^∗∗∗∗^, *P* < 0.0001; one-way ANOVA. **E,** Representative images of hypoxic zones stained with pimonidazole (brown staining) from sections of PDX tumors upon treatment with vehicle (control), 3PO, RT, and 3PO + RT. Scale bars, 100 µm. **F,** Quantification of PIMO^+^ area (%) in PDX tumors from control, 3PO, RT, and 3PO + RT-treated mice. Data are displayed as means SD. ^∗^, *P* < 0.05; ^∗∗∗^, *P* < 0.001; one-way ANOVA. **G,** Representative micrographs of γH2AX-stained sections of PDX tumors from control, 3PO, RT, and 3PO + RT-treated mice. Scale bar, 20 μm. **H,** DNA damage levels were assessed using γH2AX staining, and treated PDX tumors were immunostained and quantified for γH2AX. Data are displayed as means SD. ^∗∗∗∗^, *P* < 0.0001; one-way ANOVA.

Hypoxia is a prominent obstacle to the efficacy of radiotherapy, and tumor tissue oxygenation plays a crucial role for the efficacy of cancer treatments (45, 46). Moreover, no *in vivo* studies have been performed in human RC PDXs evaluating the use of 3PO for TVN induction, in combination with radiotherapy. Therefore, to analyze the effects of 3PO administration in combination with RT on tumor vascularization, we have performed a CD31 and αSMA co-staining with our PDX tumor samples (animal cohort from Figs. 5 and 6A). Compared with sham, tumors treated with RT alone showed a significantly reduced CD31^+^-αSMA^+^ co-stained area (vessel density), which was partially restored by concomitant treatment with 3PO, leading to comparable vessel density (Figs. 6A and B). Moreover, vessel lumen size increased when 3PO was subjected alone or added to therapy (Fig. 6C; Supplementary Fig. S5A). Further analysis showed that the vessel number decreased significantly for RT and combination therapy of 3PO and RT (Fig. 6D; Supplementary Fig. S5A). Next, tumor hypoxia was assessed by pimonidazole staining, as an indicator of tissue oxygenation [Fig. 6E; Supplementary Fig. S5B; refs. (14, 15)]. Hypoxic areas were reduced when tumors were subjected to 3PO alone or added to RT (Fig. 6F). These results reveal the TVN effects of 3PO in combination with RT in human RC. To further assess the effects of increased tumor oxygenation on the treatment of human RC with radiotherapy, we performed immunohistochemistry staining for γ-H2AX, a marker of DNA double-stranded breaks (Fig. 6G). Although animals were sacrificed 30 days after their last RT treatment, 3PO in combination with RT showed an enhanced γ-H2AX index, indicating the greatest DNA damage out of all treatment cohorts (Fig. 6H). Summarizing, we could observe the therapeutic efficacy of 3PO, through TVN in RT of human RC.

## Discussion

In order to adapt to an increased energy consumption, tumors must alter their metabolic phenotype (47). Frequently, cancer cells enhance their glycolytic activity even in the presence of oxygen, an effect described as the “Warburg effect” (48). To maintain their high glycolytic flux, multiple mechanisms might be used by cancer cells to increase their glycolytic flux, which consequently can enhance the expression of genes associated with cell motility, survival, and therapy resistance (18, 49–53). One, in particular, is the overexpression of the bifunctional enzyme PFKFB3, which plays a crucial role in glucose metabolism (54). PFKFB3 catalyzes the synthesis of F-2,6-BP, leading to intracellular accumulation of F-2,6-BP. As an allosteric activator, F-2,6-BP stimulates phosphofructokinase 1, the most potent stimulator of glycolysis, thereby meeting the requirements for increased energy metabolism of tumor cells. PFKFB3 is overexpressed in several human cancers and promotes proliferation and migration as well as suppresses apoptosis of cancer cells (55). Furthermore, PFKFB3 is known to regulate angiogenesis (35). The endothelium of tumor vessels is characterized by a highly heterogeneous and functionally abnormal structure leading to tumor hypoxia and thereby promoting tumor progression and its dissemination (11). Interestingly, TECs are characterized by an activated glycolytic phenotype and increased PFKFB3 expression. Therefore, interfering the upregulated glucose metabolism in tumor cells and TEC seems to be a promising approach to hold tumor progression and trigger TVN (14, 15). Moreover, this event seems to be possible through PFKFB3 inhibition by 3PO administration.

In this study, we have explored the effects of PFKFB3 inhibition by 3PO *in vitro* and *in vivo* using endothelial cells, colorectal cancer cells, PDOs, and PDXs. First, our results are consistent with previous studies showing that 3PO affects cell proliferation and viability of transformed cell lines in a time- and dose-dependent manner (31). Interesting, we observed that among the CRC cells tested, SW-1463 and SW-837 cells showed significantly decreased cell viability and increased LDH release as compared with the other CRC cells (Figs. 1B–E). As PFKFB3 protein expression levels do not always show a direct correlation to 3PO response (Supplementary Fig. S1B, immunoblot with CRC cell lysates), additional reasons for this phenotype upon 3PO treatment might be related with the intrinsic metabolic needs, directly dictated by the mutational status (TP53 mutation; KRAS mutation; CpG island methylated phenotype) and by its transit-amplifying undifferentiated phenotype, which could also influence its susceptibility to 3PO (32, 33). Importantly, we cannot exclude the intrinsic differences between cell lines in the expression of PFKFB3 and intracellular F2,6BP levels, which cannot always be directly correlated with glycolytic flux (56). Regarding the selectivity of 3PO cytotoxicity on normal, nontransformed cells, we observed that HUVECs and RPE cells well tolerated the lower *in vitro* doses, compared with most of the cancer cells. Therefore, we hypothesized that the used normal, nontransformed cells were able to shift more easily from glycolysis to oxidative phosphorylation (OXPHOs) than transformed cells, preventing them from suffering massive cell death.

Next, we showed that treatment of different CRC cell lines with a low dose of 3PO caused a reduction in tumor cell migration and invasion, indicating that blocking glycolysis in cancer cells is associated with cytoskeletal rearrangements (18). Simultaneously, we observed reduced expression of *PAXILLIN*, *CDC42*, and *WASF-1*. Moreover, consistent with the fact that HCT-116 exhibited no reduction in migration by PFKFB3 inhibition, there were no alterations in the expression of those genes in this cell line. Differences in its mutational status might be responsible for the observed results, as HCT-116 is highly hypermutated and microsatellite instable, as opposed to HT-29, SW-837, and SW-1463, which are nonhypermutated and microsatellite stable (32, 33, 57). This hypothesis is supported by recent proteomic data showing glycolytic differences between microsatellite instable and stable CRCs, which may have not only paracrine but also autocrine effects (58). It is noteworthy that from all four cell lines used in the invasion assay, SW-1463 cells were the most susceptible to 3PO effects on viability, and therefore we cannot exclude that its reduction in invasion is also related to 3PO cytotoxic effects. Furthermore, we have observed that endothelial spheroids subjected to low doses of 3PO show reduced sprouting and vessel-like formation and that this effect could be due to reduced endothelial proliferation. Nevertheless, the effects on the tip cell phenotype cannot be excluded, as it has been suggested that PFKFB3 is also involved in this process (59).

Corroborating the idea that 3PO treatment would efficiently disturb glycolysis in cancer cells, and that CRT resistance could be overcome upon inhibition of glycolysis, we observed a shift in the transcriptomic metabolic flux of patient-derived tumor organoids toward oxidative phosphorylation, which denotes a possible compensatory mechanism to account for energy deprivation caused by reduced glycolysis (60, 61). Additionally, a subunit of complex I, the largest complex in mitochondrial respiratory chain, NDUFB6 expression is enhanced after treatment with 3PO, and as previously shown by Shi and colleagues (62), CRC cells deficient in complex I exhibited enhanced radiotherapy resistance, along with increased glycolysis. Moreover, the gene set “GOBP Extracellular Matrix Assembly” was found most enriched in the control group, reinforcing previous findings that glycolysis can affect extracellular composition and therefore further influence cell proliferation, adhesion, and migration (18, 34, 63). Remarkably, by reviewing the most relevant gene sets of 3PO-induced genes on CRC in the protein atlas (NDUFB6, NDUFAB1, NDUFA4, CDC7, CDKN3, PLK4; ref. 64), we found that the increased expression of these proteins correlated with a better prognosis (Supplementary Fig. S6).

Next, we hypothesized that RT resistance could be overcome upon inhibition of glycolysis. Other studies have shown that CRC cells were sensitive to glucose deprivation through PFKFB3 inhibition (18, 65). However, in these studies, a radiosensitizing effect through PFKFB3 inhibition on CRC cells remained unclear. Our results demonstrate that PFKFB3 inhibition by 3PO increases radiation-induced cell death of CRC cells while reducing cancer cell migration and cancer cell invasion during RT *in vitro*. Previously, it has been suggested that 3PO could induce changes in cell cycle (31) and that cancer cells arrested at the G2M phase would exhibit increased sensitivity to irradiation (66). Interestingly, at the used lower 3PO concentrations, we did not observe changes in cell cycle upon 3PO treatment (Supplementary Fig. S7A–S7C), which led us to speculate that these effects were associated (i) with direct changes in the expression of migration/invasion-related genes and (ii) with reduced ability to buffer increased radiation-induced radicals (ROS) upon a shift to metabolic flux toward oxidative phosphorylation, possibly associated with reduced accumulation of glycolytic products, such as glutathione and pyruvate (67).

Considering our *in vitro* results, we decided to evaluate the treatment response of human RC PDXs to 3PO *in vivo*, as it has been already shown that 3PO administration at lower doses does not cause severe systemic effects in mice (15, 31, 68). Previously, we showed that in a mouse model of murine melanoma, 3PO treatment at low concentrations triggered TVN (14). Now, using human patient-derived RC xenografts, we demonstrate that tumors treated with concomitant administration of 3PO and RT exhibited reduced hypoxia, suggesting that also in RC, the inhibition of PFKFB3 by 3PO leads to a similar outcome. This finding was supported by enlarged vessel lumina through CD31 staining upon 3PO treatment and changes in tumor vessel density. Interestingly, this result also suggests that in mice, at least partially, TVN can be achieved without T cells’ mediation, a finding previously discussed by Lian Tian and colleagues (69). Of clinical relevance, further evaluation of 3PO effects at low doses on immune cell function will be necessary, as many linages like T cells require glycolysis for their proper activation (70).

Regarding tumor response to RT, we show that administration of low doses of 3PO in combination with RT resulted in extensive tumor necrotic areas when compared with control groups. This effect was associated with increased tumor DNA damage and cell death, evidenced by both increased H2AX phosphorylation and TUNEL-positive tumor cells. These results are in line with the report that PFKFB3 inhibition can directly affect DNA damage repair and homologous recombination upon radiation (71). Moreover, increased DNA damage can also be attributed to an increase in tumor oxygenation levels due to TVN, which further triggers ROS-mediated tumor cell death. Importantly, we have not observed macroscopic differences in tumor sizes between single RT and combined 3PO + RT cohorts (Fig. 5B). This result might be related to pronounced tumor sensitivity to RT and/or the dose of administrated RT. Thus, further studies using different doses of radiation will be necessary to evaluate the contribution of 3PO for combinatory chemotherapy treatments with and without radiation.

Currently, no PFKFB3 inhibitors are used in the clinic alone or in combinational approaches. Hence, our results indicate that PFKFB3 inhibition by 3PO could be a potential therapeutic target in human RC, reducing cancer cell survival *in vitro* and inducing TVN *in vivo*, which ultimately may improve the currently established therapy to increase the rate of clinical complete responses.

## Supplementary Material

Supplementary Fig.1 Supplementary Fig.1

Supplementary Fig. 2 Supplementary Fig. 2

Supplementary Fig. 3 Supplementary Fig. 3

Supplementary Fig. 4 Supplementary Fig. 4

Supplementary Fig. 5 Supplementary Fig. 5

Supplementary Fig. 6 Supplementary Fig. 6

Supplementary Fig. 7 Supplementary Fig. 7
