# Supplementary figures and images for: Tumor Vessel Normalization via PFKFB3 Inhibition Alleviates Hypoxia and Increases Tumor Necrosis in Rectal Cancer upon Radiotherapy
Source: Cancer Res Commun. 2024 Aug 9;4(8):2008–24. doi: 10.1158/2767-9764.CRC-24-0077 (PMC11310748; doi:10.1158/2767-9764.CRC-24-0077)

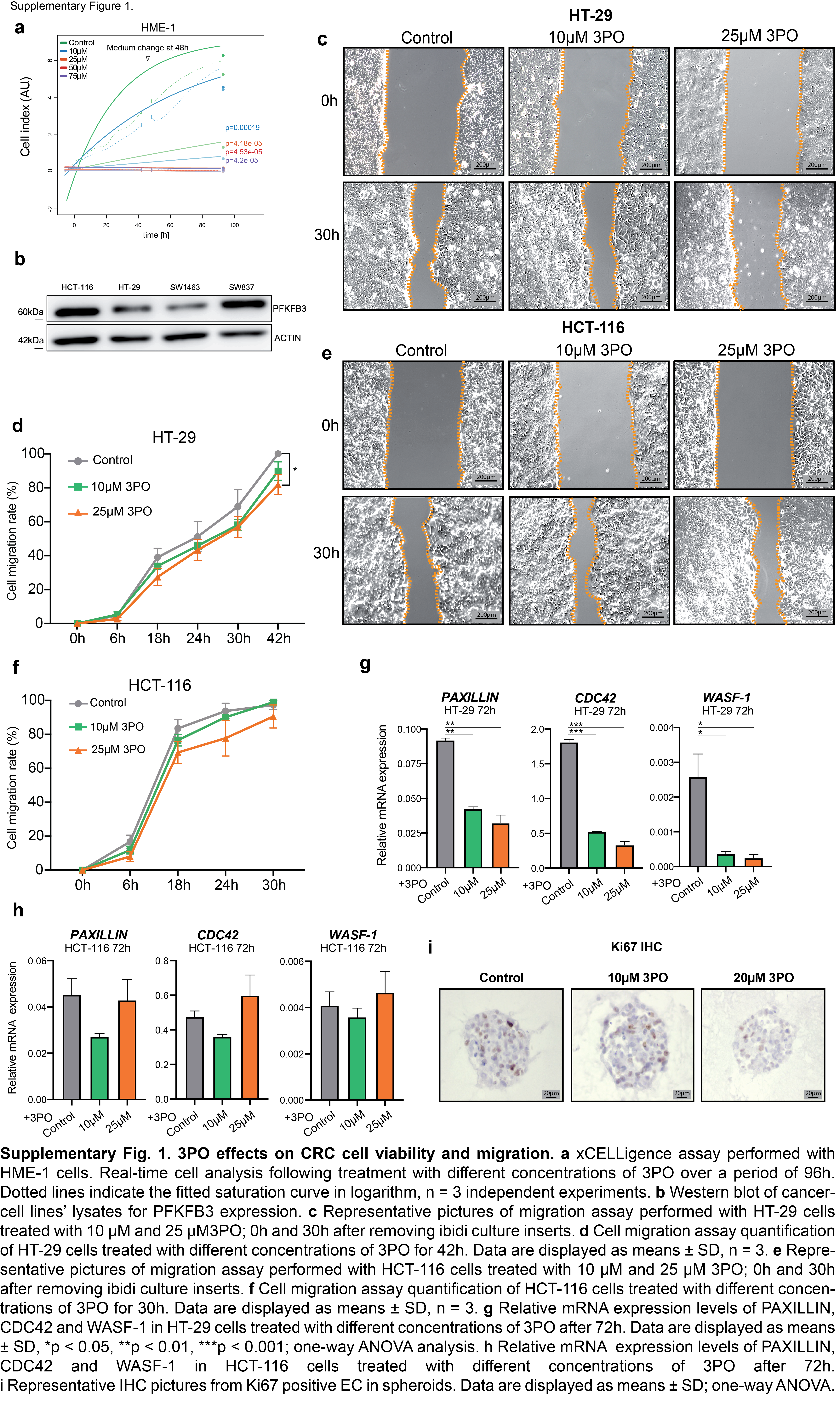

Supplement: Supplementary Fig.1 [file crc-24-0077_supplementary_fig.1_suppsf1.png]

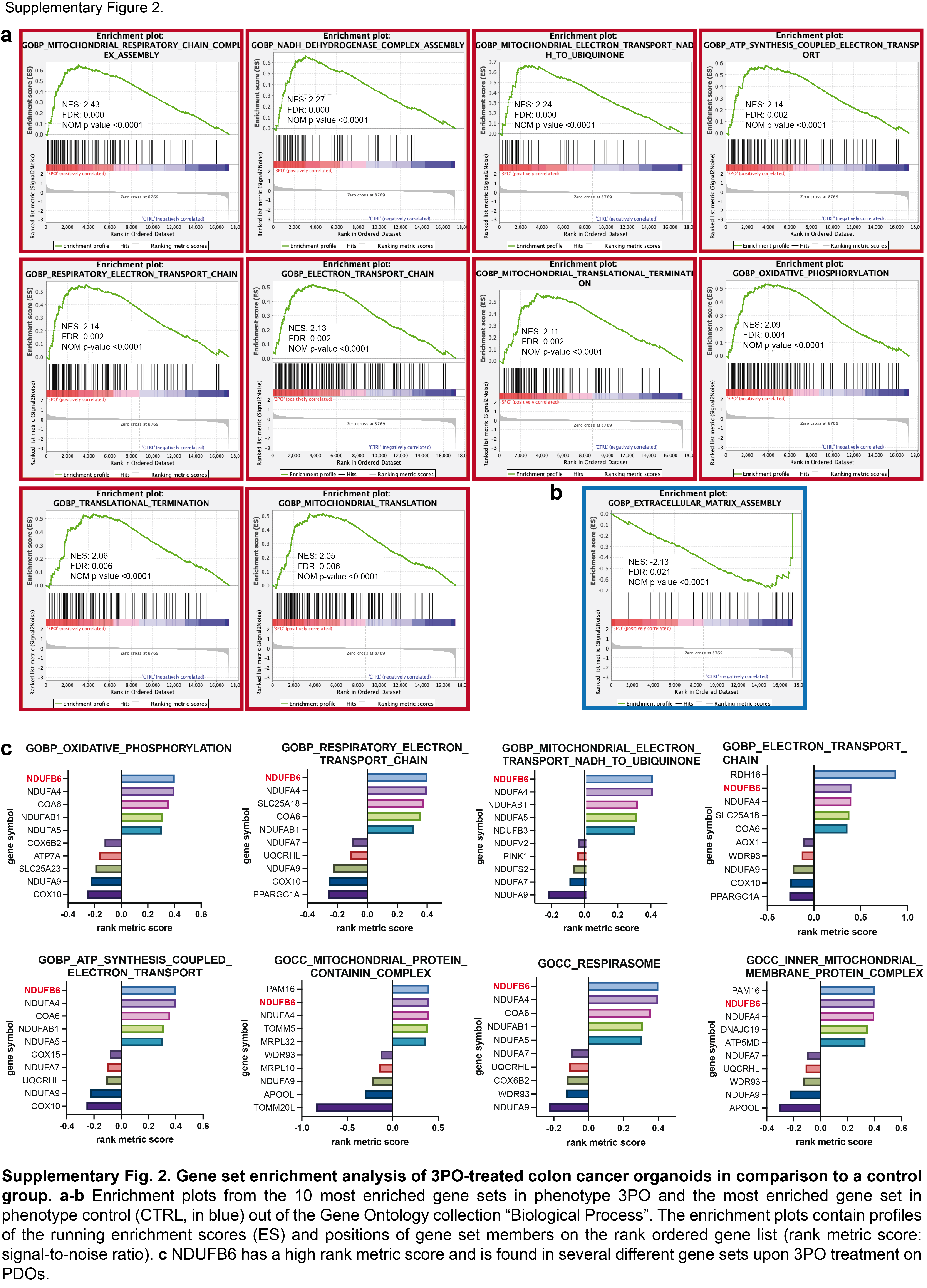

Supplement: Supplementary Fig. 2 [file crc-24-0077_supplementary_fig.2_suppsf2.png]

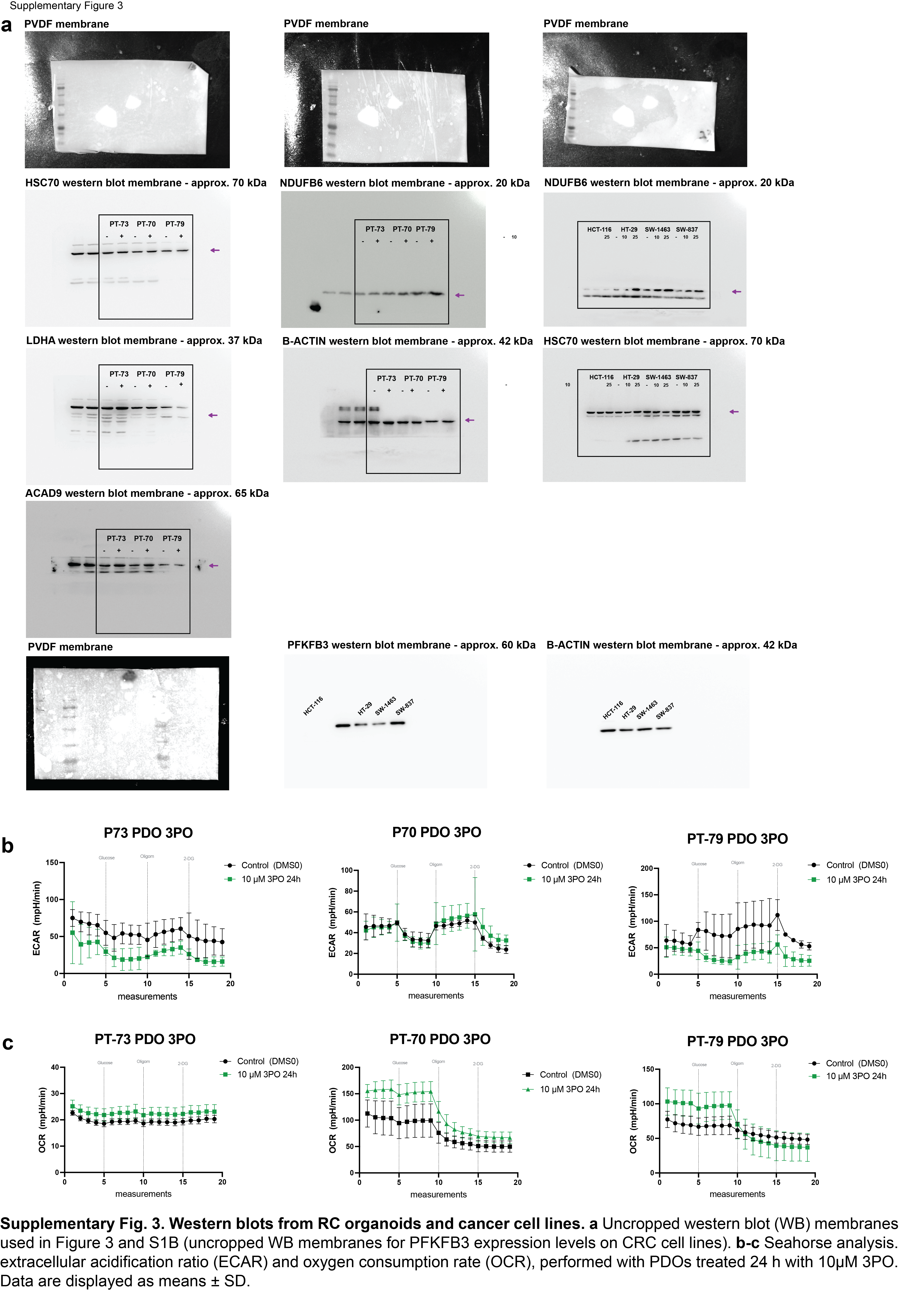

Supplement: Supplementary Fig. 3 [file crc-24-0077_supplementary_fig.3_suppsf3.png]

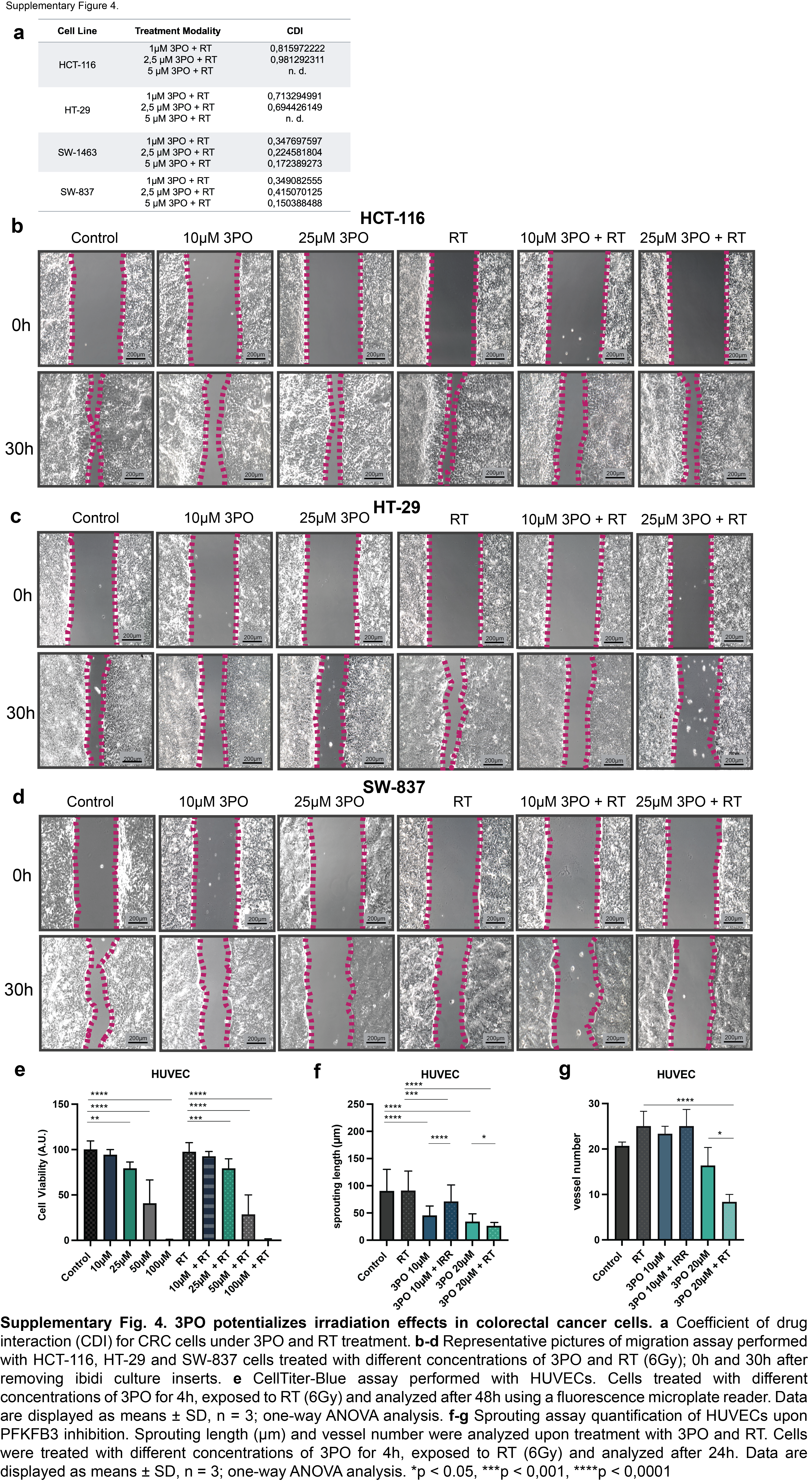

Supplement: Supplementary Fig. 4 [file crc-24-0077_supplementary_fig.4_suppsf4.png]

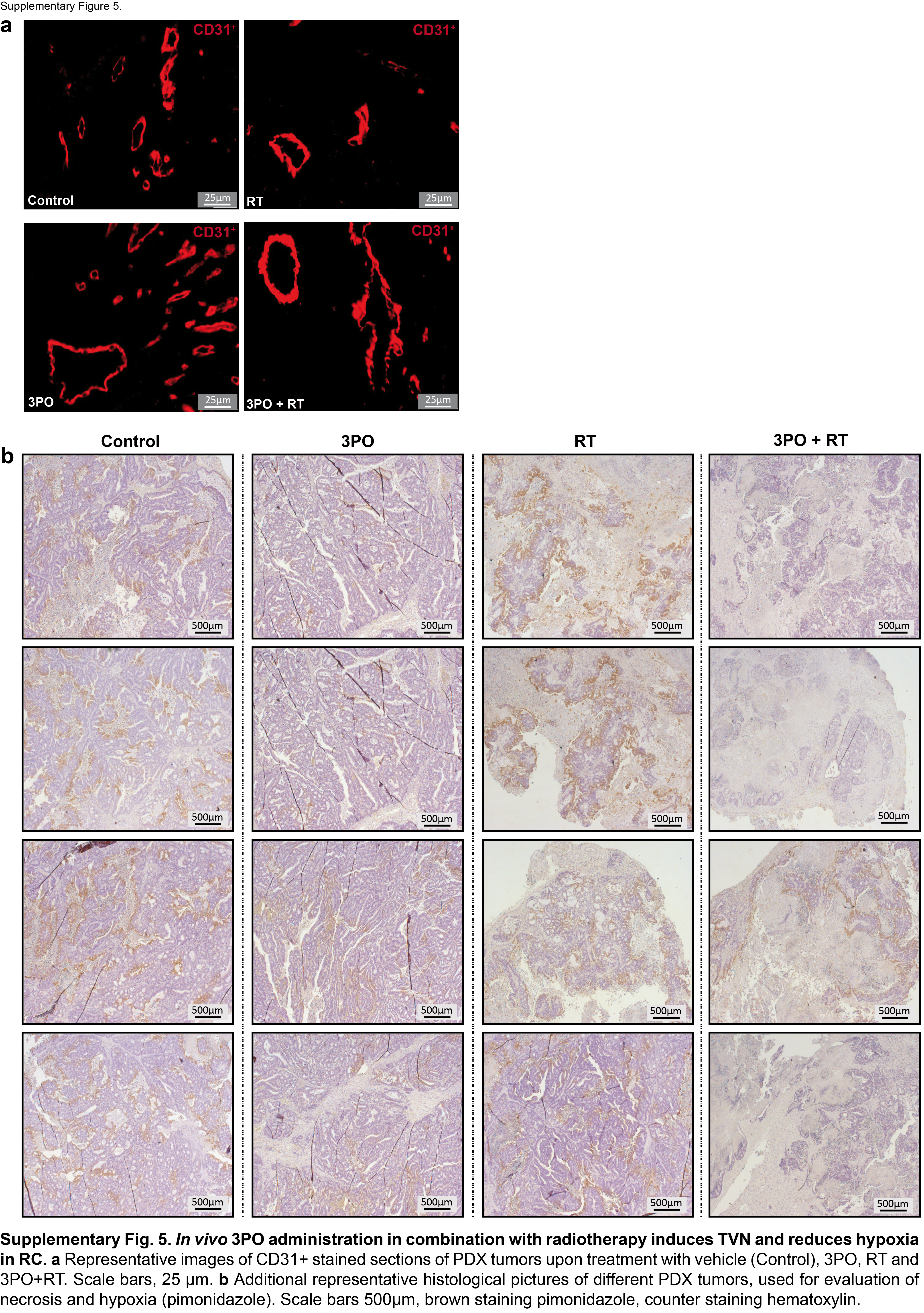

Supplement: Supplementary Fig. 5 [file crc-24-0077_supplementary_fig.5_suppsf5.png]

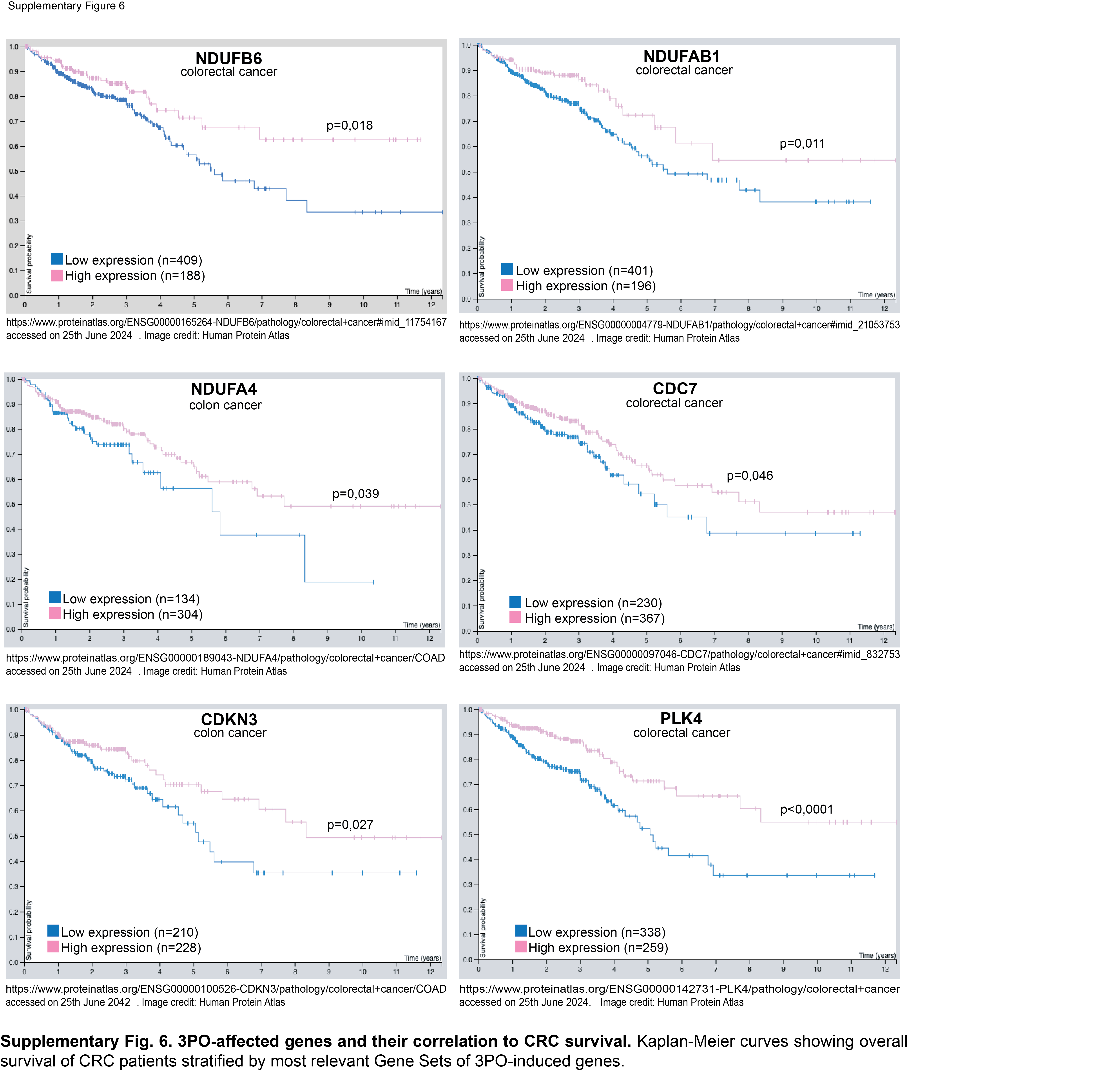

Supplement: Supplementary Fig. 6 [file crc-24-0077_supplementary_fig.6_suppsf6.png]

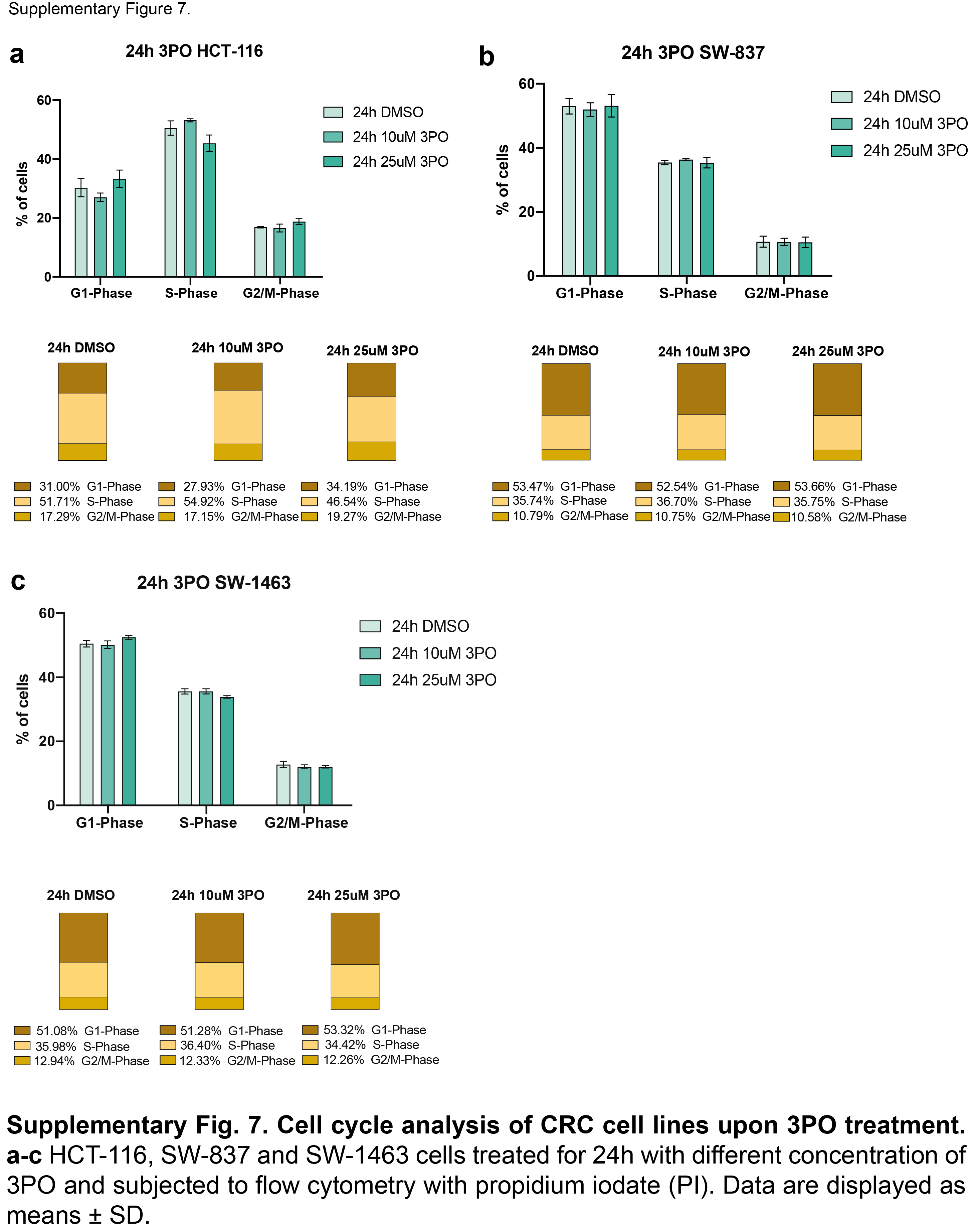

Supplement: Supplementary Fig. 7 [file crc-24-0077_supplementary_fig.7_suppsf7.png]
